# Transepithelial corneal crosslinking in treatment of progressive keratoconus: 12 months’ clinical results

**DOI:** 10.12669/pjms.333.11907

**Published:** 2017

**Authors:** Bushra Akbar, Rana Intisar-ul-Haq, Mazhar Ishaq, Sabahat Arzoo, Kashif Siddique

**Affiliations:** 1 Dr. Bushra Akbar, MBBS. Department of Ophthalmology, Armed Forces Institute of Ophthalmology, AFIO, Rawalpindi, Pakistan; 2 Dr. Rana Intisar ul Haq, MBBS, DO, MCPS, FCPS. Department of Ophthalmology, Armed Forces Institute of Ophthalmology, AFIO, Rawalpindi, Pakistan; 3 Dr. Mazhar Ishaq, MBBS, FRCS Ed, FRCOPHTH, FCPS. Department of Ophthalmology, Armed Forces Institute of Ophthalmology, AFIO, Rawalpindi, Pakistan; 4 Sabahat Arzoo, BSC (Hons.) Optometry, Orthoptics. Department of Ophthalmology, Armed Forces Institute of Ophthalmology, AFIO, Rawalpindi, Pakistan; 5 Kashif Siddique, M.Sc (Biostatistics). King Salman Armed Forces Hospital, Tabuk, KSA Academic Affairs (Research Unit), Saudi Arabia

**Keywords:** Transepithelial CXL, Pakistani population, Thin corneas, Accelerated UVA irradiation, Progressive keratoconus

## Abstract

**Objective:**

The purpose of this study was to evaluate the safety and efficacy of transepithelial corneal collagen cross linking (TE-CXL) with modified riboflavin and accelerated UVA irradiance in thin corneas with pachymetry less than 400 microns at thinnest point, untreatable by epithelium off corneal collagen cross linking (CXL) in adult Pakistani population with progressive keratoconus.

**Methods:**

This quasi experimental study included twenty six eyes of 26 patients with progressive keratoconus who underwent accelerated transepithelial CXL in Armed forced institute of ophthalmology with 12 months follow up. Modified riboflavin, ParaCel ((riboflavin 0.25%, Benzalkonium chloride, EDTA, Trometamol, hydroxypropyl methylcellulose) and vibeX Xtra (riboflavin 0.25%) (Avedro, USA)) were applied to cornea in two stages. Uncorrected and Corrected Distant Visual Acuities (UDVA, CDVA), spherical equivalent (SE), astigmatism, pachymetry at thinnest point (Pachy thin), apex keratometry (Kmax), simulated and steep keratometry (Sim K, steep K) were measured at baseline and at 3, 6 and 12 months post operatively. The cornea was then exposed to accelerated UVA irradiance of 9mW/cm^2^ for 10 min (total dose 30 mW/cm^2^).

**Results:**

The mean age of the patient was 24.54±5.16 years. UDVA, CDVA, SE, astigmatism significantly improved at all postoperative test points (*p*=0.000, 0.004, 0.000, 0.004 respectively). Kmax and pachy thin were significantly reduced over baseline at 1 year (*p*=0.000, 0.004 respectively). Topographic indices Sim K and steep K did not show significant changes. No intra or post-operative complications were reported.

**Conclusion:**

Transepithelial accelerated CXL with modified riboflavin is a safe and effective procedure which halt disease progression in thin corneas with progressive keratoconus.

## INTRODUCTION

Corneal Collagen Cross Linking (CXL) is a novel and promising technique known to halt the progression of keratoconus and secondary keratectasia by increasing the biomechanical strength of the cornea, deferring the necessity of corneal grafts.^1^ It involves the photo activation of riboflavin with Ultra violet A (UVA) radiation, that unfolds a series of photochemical reactions inducing inter and intra fibrillary cross links in the corneal stromal lamellae.^2^ This increases the tensile strength of cornea preventing further thinning and deformation of corneal profile,^3^ deterioration of vision and offers functional improvement to some degree with minimal complications.^4^

The classical CXL protocol described by wollensek required debridement of the central 7 to 9 mm of corneal epithelium and a 30 minute pre-soak with riboflavin-5-phosphate and 20% dextran t-500 followed by continuous wave illumination of 3 mW/cm^2^ irradiance of 365 nm UVA for 30 minutes.^2^ It was associated with an increased incidence of post-operative pain, infection stromal haze and epithelial healing problems.^2^,^5^ This warranted the modification of riboflavin solutions, treating protocols, UVA exposure and irradiation duration, in terms of transepithelial and accelerated protocols.

Recently transepithelial cross-linking, gained rounds by leaving epithelium intact, improving patient comfort and minimizing the risk of infection associated with epithelial removal.^6^ However, it’s efficacy remained controversial as epithelial barrier limit the depth and amount of cross-linking, evidenced as shallow and uneven distribution of the keratocytes apoptosis on confocal microscopy^7^ and ectatic progression in many clinical trials as compared to epithelium off CXL.^8^,^9^ The stratified corneal epithelium with tight junctions presents as a lipophilic barrier to the absorption of the hydrophilic riboflavin macromolecule. Standard riboflavin formulations were modified with addition of EDTA, benzalkonium chloride (BAC), trometamol, which loosen up the epithelial junctions, enhancing the permeability and riboflavin diffusion across the epithelium and superficial stroma.^6^,^10^ However cumulative dose of BAC in ParaCel alone may result in damage to the superficial corneal epithelial cells^11^ as its effect is concentration and dose dependent.^12^ A recent two stage application of initial ParaCel containing BAC followed by BAC and dextran free vibeX Xtra combined with accelerated UVA irradiance protocols (total energy dose of 5.2j/cm^2^ or 7.2 j/cm^2^), reduced riboflavin soak time and promoted epithelial integrity,^13^ maximizing patient comfort and disease stabilization. To the best of our knowledge, It is first study on adult Pakistani population of progressive keratoconus investigating the efficacy and safety of two stage application of ParaCel and vibeX Xtra on thin corneas, combined with accelerated protocol (9mwatt/cm^2^ for 10 min, total dose intensity 5.4j/cm^2^) of UVA irradiance. We aimed to report our 1 year clinical results of combined ParaCel and vibeX Xtra assisted transepithelial accelerated CXL on thin keratoconic corneas in adult Pakistani population with progressive keratoconus.

## METHODS

This quasi experimental study was conducted at Armed Forces Institute of Ophthalmology from April 2015 to September 2016 after approval of Hospital ethical review committee. Twenty six eyes of 26 patients of progressive keratoconus, who underwent Combined ParaCel and vibeX Xtra assisted transepithelial CXL with a follow up of at least 12 months were included in this study. An informed consent was obtained from all patients. Inclusion criteria was documented progression of keratoconus in patients between 16 to 36 years of age, and corneal pachymetry <400 microns and >375 microns at thinnest point, considered unfit for epithelium off CXL. Progressive keratoconus was defined as an increase in central corneal astigmatism of >1.00D or an increase of 1.00D in maximum cone apex curvature documented over a 6 months duration. Exclusion criteria included eyes with history of previous ocular surgery, secondary keratectasia, severe keratoconjunctivitis sicca, active ocular infection, corneal melting, epithelial healing problems, systemic autoimmune disorders, pregnancy and lactation. All contact lens wearers were advised to discontinue contact lens 2 weeks prior to baseline evaluation. CXL procedure and every post-operative follow up examination.

All patients underwent comprehensive ophthalmic evaluation at baseline and three, six and 12 months post operatively including uncorrected and corrected distant visual acuities [(UDVA, CDVA, and Snellen visual acuity converted to logMAR notation)], SE, topographic astigmatism, slit lamp biomicroscopic and dilated fundus exam. Topographic indices sim K, steep K, Kmax and pachy thin were analyzed on a dual scheimpflugh corneal topography (Galilie G4). The primary outcome measure was disease stabilization defined as an increase of no more than 1 dioptre in Kmax over baseline at 12 months follow up. Worsening of Kmax over 1 dioptre was considered as progression of keratoconus and ineffectiveness of TE-CXL.

The special riboflavin solutions used were ParaCel (riboflavin 0.25%, benzalkonium chloride, EDTA, Trometamol, hydroxypropyl methylcellulose, phosphate buffered saline solution) and vibeX Xtra (riboflavin 0.25%, phosphate buffered saline solution) (Avedro, USA).

### Surgical technique

TE –CXL was carried out as day care procedure in an operating room under topical anaesthesia with 0.05% proparacaine (alcaine) eye drops. Patient was placed in a supine position. Surgical field was prepared and eyelid speculum was inserted using standard clinical techniques. Corneal epithelium was left intact. ParaCel was initially applied to completely cover the cornea and this procedure was repeated every 90 seconds for a total of 4 minutes. Cornea was then rinsed completely with vibeX Xtra. VibeX Xtra was then applied completely covering the cornea at a rate of one drop every 90 seconds for a total of 6 minutes. UV A treatment was initiated for 10 minutes at an irradiance of 9mW/cm^2^ (accelerated protocol) at 55mm from the cornea, with a total energy of 5.2j/cm^2^ (CCL VARIO, PESCHKE Trade GmbH, Huenenberg Switzerland). Cornea was then completely rinsed with balanced salt solution. Eyelid speculum was removed. Patient was advised to refrain from eye rubbing. Antibiotic moxifloxacin (Vigamox 0.05%, Alcon) and lubricants hypromellose, (Tear Naturale II, Alcon) were advised for one week. Corticosteroids fluorometholone 0.1% (FML0.1%, Allergan) eye drops were continued for two weeks. Bandage contact lens (Interojo, Korea) was applied which was removed on first postoperative day.

### Statistical analysis

Data analysis was done by using SPSS version 20. Quantitative variables were expressed as mean±standard deviation. After normality testing Friedman ANOVA test was applied to evaluate changes in different parameters from baseline to follow up time points of 3, 6 and 12 months post TE-CXL. *p*<0.05 with 95% CI was considered as statistically significant.

## RESULTS

The mean age of patients was 25.46±5.16 (range 18 to 36 years). There were14 males and 12 females in our study. Table-I: The mean UDVA at baseline was 1.31.±0.45 which significantly improved at postoperative test points of 3 and 6 months with a final UDVA of 0.97±0.51 at 12 months (*p*=0.000). The mean CDVA did not show any significant improvement. The primary outcome measure, disease stabilization, was achieved in 27(90%) eyes and only 3(10%) eyes showed progression predefined as increase of more than 1.00 dioptre in Kmax over the baseline at 12 months follow up. Table-III. The mean Kmax 62.49±9.55D at baseline showed significant flattening to 60.83±9.49 at 1 year follow up *(p=*0.000). SE, astigmatism, Pachy thin showed a significant reduction at 1 year follow up as compared to preoperative examination (*P*=0.000, 0.004, 0.000 respectively). Table-II & III: Topographic indices (simulated K and steep K) showed reduction over the postoperative test points, however it was not statistically significant (*p*=0.083, 0.133 respectively) Table-III: No peroperative or postoperative complications were documented.

**Table-I:** Demographic data of patients.

*n*	*26 eyes*
Age (Years)	25.46± 5.16 (18-36 Years)
Male	14
Female	12

**Table-II:** Changes in refractive parameters at baseline and post-operative follow up.

	*Preoperative*	*Post-operative*			*p-value*

		*3 Months*	*6 Months*	*12 Months*	
VA (Log MAR)	1.31±0.45	1.25±0.43	1.06±0.49	0.97±0.51	0.000^(a)^*
BCVA (Log MAR)	0.51±0.28	0.54±0.28	0.45±0.32	0.51±0.29	0.044^(a)^*
Spherical Equivalent	-5.89±2.44	-5.54±2.90	-4.52±2.27	-4.52±3.19	0.000^(a)^*
Astigmatism	-5.1±3.66	-3.7±2.52	-4.37±3.20	-3.00±1.77	0.004^(a)^*

***Note:***

(*) p-value < 0.05, (a): Friedman ANOVA Test.

**Table-III:** Changes in topographic parameters and pachymetry at baseline and post-operative follow up.

	*Preoperative*	*Post-operative*			*p-value*

		*3 Months*	*6 Months*	*12 Months*
Steep K	56.34±7.28	55.88±6.9	55.27±6.60	54.44±6.32	0.133^(a)^
Sim K	51.14±4.07	51.20±4.58	51.07±4.49	50.68±4.29	0.083^(a)^
Pachy Thin	395.15±14.85	393.34±15.76	386.88±13.72	384.61±17.77	0.000^(a)^*
K Max	62.49±9.55	61.39±9.43	61.72±9.47	60.83±9.49	0.000^(a)^*

***Note:***

(*) p-value < 0.05, (a): Friedman ANOVA Test

## DISCUSSION

Our current study showed significant improvements in UDVA, SE, astigmatism and reduction in Kmax and Pachy thin with ParaCel and vibeX Xtra assisted accelerated transepithelial CXL at 12 months. The success rate in terms of disease stabilization was 90% at 1 year and only 3 (10%) eyes exhibited continued ectatic progression.

Transepithelial method is a promising recent modification in CXL, which offers obvious advantages of eliminating the inconvenience of conventional method in terms of pain free, faster visual recovery and decreased risk of post-operative infection by leaving the epithelium intact.^7^ The barrier function of the intact epithelium also limit the key components of effective cross linking like optimal homogenous riboflavin saturation and incident UVA light penetration.^13^-^15^ ParaCel (avedro), is a dextran-free, hypoosmolar new transepithelial riboflavin formulation that contains epithelial permeability enhancers like BAC, EDTA to facilitate penetration through intact epithelium. However chronic use of BAC containing eye drops, may result in superficial corneal epithelial toxicity, similar to dry eye syndrome due to its cumulative dose^6^,^11^ as BAC effects are both concentration and duration dependent.^14^ Therefore, two-stage riboflavin application model minimized the duration of BAC exposure offering desirable balance between permeability enhancement and epithelial preservation. Initial four minute soak with ParaCel loosen epithelial junctions and ensure adequate dose of riboflavin. VibeX Xtra 0.25%, BAC-free riboflavin solution then flush the ParaCel and provides an additional six minutes of riboflavin soaktime.^13^ In our study we neither prefix the riboflavin application with 30 min proxymetacaine chloride, as demonstrated by Kir et al,^16^ Nor did we used a corneal epithelial trephine for riboflavin soak like Zhang et al for enhanced permeability.^17^ We here adhered to manufacturer guidelines, where ParaCel and vibeX Xtra were sufficiently applied to cornea with eye lid speculum on. The original CXL protocol of 3mW/cm^2^ irradiance of 365 nm UVA for thirty min duration with a total dose intensity of 5.4j/cm^2^ required 60 minutes for complete procedure. Equally effective and accelerated protocols were introduced to shorten the duration of treatment based on Bunsen and Roscoe law of reciprocity that states, photochemical biological effect is proportional to the total energy dose delivered regardless of the applied irradiance and time. High intensity accelerated protocols such as 9mW/cm^2^ for 10 minutes,18 mW/cm^2^ for 5 mintes,30 mW/cm^2^ for 3minutes, with a total dose of 5.4j/cm^2^ and 30 mW/cm^2^ for 4minuters and 45 mW/cm^2^ for 2 min 40 sec with a total energy dose of 7.2j/cm^2^ gave comparable results to conventional method in treatment of progressive keratoconus.^18^,^19^ We used accelerated protocol of 9mW/cm^2^ for 10 minutes, total dose of 30 mW/cm^2^, 5.4j/cm^2^ for UVA irradiance, unlike manufacturer guidelines of 45mwatt/cm^2^ owing to max 30 mW/cm^2^ limitation of our CCL-VARIO corneal cross linking device. Clinical stabilization achieved in current study was still comparable to stability achieved in 45mwatt/cm^2^ high intensity accelerated TE-CXL studies.^16^,^17^

Zhang et al reported a significant gain in mean UDVA from 1.02±0.55 to 0.58±0.34 at 12 months post 45mwatt/cm^2^ high intensity accelerated that closely relate to our study results of improvement of mean pre-op UDVA from 1.31±0.45 to 0.97±0.51 at 1 year post-operative follow-up exam.^17^ The fattening of 1.66 Dioptres of Kmax achieved in our study may attribute to high pre Kmax Of 62.49 D which tends to flatten more with CXL.^8^ Spadea also described a flattening of 3.09 Dioptres of mean K max of 61.30 at 1 year follow up compared to Kir et al and Zhang et al. where significant reduction was not achieved with a pre K max of 54.6 and 55.47 Dioptres respectively.^16^,^17^,^20^ Some investigators reported stability^8^,^9^ while others found worsening of topographic indices, and continued progression of the disease.^21^,^22^ This conflicting outcomes can be attributed to variable treating protocols and riboflavin solutions used by investigators on different populations.

The Pachymetry at thinnest point on dual scheimpflugh corneal topography decreased significantly from baseline p<0.000 at 1 year follow up visit in agreement with previous studies.^8^,^9^ These changes in corneal thickness are considered secondary to the lamellar remodeling of corneal stroma after CXL.^23^

The relatively thin corneas, high preop Kmax, and younger age of the patients, in our study indicates an advanced stage of disease with a likelihood for progression after an initial stability at 12 months as Keratoconus progression does not exhibit a linear trend over time. Thus a further long term follow up with updates on clinical stability is recommended. The unavailability of confocal microscopy to document structural changes, depth and homogeneity of keratocytes apoptosis; lamellar remodeling and endothelial function is the major limitation of our study. Moreover lack of control groups with no intervention makes an additional limitation. However it was not considered ethical to leave keratoconus patients untreated due to the progressive nature of disease. We strongly recommend studies with large sample size, long term follow up and variable UVA irradiance protocols to retest and compare our results with international trials for optimizing TE CXL protocols for our population.

## CONCLUSION

In conclusion, two stage ParaCel and vibeX Xtra assisted accelerated TE CXL is a safe and effective procedure which halt keratoconus progression in thin corneas, at 12 months follows up in Pakistani population offering an additional benefit of shorter surgical time, increased patient comfort and faster visual recovery.

### Authors` Contribution

**BA:** Conception of design, acquisition, interpretation, analysis of data, manuscript drafting and critical revision of final draft before submission.

**RIH:** Study design conception, critical revision of manuscript, final approval of draft for publication, agreed to be responsible for accuracy and integrity of any part of work.

**MI:** Critical revision of manuscript, final approval of draft, agreed to be responsible for accuracy and integrity of any part of work.

**PC:** Data collection, interpretation of data and manuscript drafting.

**KS:** Data interpretation and analysis, manuscript drafting.
